# WHO research agenda on the role of the institutional safety climate for hand hygiene improvement: a Delphi consensus-building study

**DOI:** 10.1136/bmjqs-2024-017162

**Published:** 2024-10-04

**Authors:** Ermira Tartari, Julie Storr, Nita Bellare, Claire Kilpatrick, Maryanne McGuckin, Mitchell J Schwaber, Didier Pittet, Benedetta Allegranzi

**Affiliations:** 1 Infection Prevention and Control Technical and Clinical Hub, Department of Integrated Health Services, World Health Organization, Geneva, Switzerland; 2 Faculty of Health Sciences, University of Malta, Msida, Malta; 3 Retired or Former, University of Pennsylvania Faculty, Philadelphia, Pennsylvania, USA; 4 National Center for Infection Control, Israel Ministry of Health, Jerusalem, Israel; 5 Faculty of Medicine, University of Geneva, Geneva, Switzerland

**Keywords:** infection control, safety culture, healthcare quality improvement

## Abstract

**Background:**

Creating and sustaining an institutional climate conducive to patient and health worker safety is a critical element of successful multimodal hand hygiene improvement strategies aimed at achieving best practices. Repeated WHO global surveys indicate that the institutional safety climate consistently ranks the lowest among various interventions.

**Methods:**

To develop an international expert consensus on research agenda priorities related to the role of institutional safety climate within the context of a multimodal hand hygiene improvement strategy, we conducted a structured consensus process involving a purposive sample of international experts. A preliminary list of research priorities was formulated following evidence mapping, and subsequently refined through a modified Delphi consensus process involving two rounds. In round 1, survey respondents were asked to rate the importance of each research priority. In round 2, experts reviewed round 1 ratings to reach a consensus (defined as ≥70% agreement) on the final prioritised items to be included in the research agenda. The research priorities were then reviewed and finalised by members of the WHO Technical Advisory Group on Hand Hygiene Research in Healthcare.

**Results:**

Of the 57 invited participants, 50 completed Delphi round 1 (88%), and 48 completed round 2 (96%). Thirty-six research priority statements were included in round 1 across five thematic categories: (1) safety climate; (2) personal accountability for hand hygiene; (3) leadership; (4) patient participation and empowerment and (5) religion and traditions. In round 1, 75% of the items achieved consensus, with 9 statements carried forward to round 2, leading to a final set of 31 prioritised research statements.

**Conclusion:**

This research agenda can be used by researchers, clinicians, policy-makers and funding bodies to address gaps in hand hygiene improvement within the context of an institutional safety climate, thereby enhancing patient and health worker safety globally.

**WHAT IS ALREADY KNOWN ON THIS TOPIC**

- Improving the institutional safety climate is a key element of the WHO multimodal strategy for hand hygiene improvement in healthcare settings.
- Yet, available evidence on the precise relationship between the institutional safety climate and hand hygiene improvement remains suboptimal.

**WHAT THIS STUDY ADDS**

- By engaging a multidisciplinary group of experts, we generated a list of priorities for a global research agenda to address the importance of the institutional safety climate for hand hygiene improvement.

**HOW THIS STUDY MIGHT AFFECT RESEARCH, PRACTICE OR POLICY**

- Our findings will help to guide policy-makers and funding bodies in decision-making, prioritisation and funding allocation for a multimodal hand hygiene improvement strategy.
- They will also guide researchers and clinicians in designing research questions and protocols to ensure that future research addresses important areas for investigation related to how a safety climate can be most effectively achieved in healthcare facilities and how it contributes to hand hygiene improvement, with the ultimate objective to improve patient safety and quality of care.

## Introduction

 WHO has served as a global leader in advocating for and supporting hand hygiene as a critical preventive measure against infectious pathogens for almost two decades, particularly in healthcare settings.^1 2^ However, compliance with hand hygiene recommendations remains suboptimal globally, with an average compliance level of 59.6% in intensive care units and significant disparities between high- and low-income countries (64.5% vs 9.1%).^3 4^ This ongoing challenge has been the focus of numerous quality improvement programmes.^5^ To achieve behavioural change and enhance hand hygiene practices among health workers, it is essential to implement institution-wide strategies that promote and support a positive organisational culture and a heightened patient safety climate and to continue to use hand hygiene as a quality indicator in healthcare.

Building on previous research, the WHO guidelines on the core components for effective infection prevention and control (IPC) programmes^6^ recommend the use of a multimodal improvement strategy (MMIS) that has already proven to be highly effective.^1^ The MMIS comprises five elements: (1) system change, involving the improvement of human resources, equipment availability and infrastructure at the point of care to facilitate best practices; (2) training and education for health workers and key stakeholders; (3) evaluation and feedback, involving the monitoring of practices, processes and outcomes and the provision of timely feedback; (4) communications and reminders in the workplace and (5) an institutional safety climate.^1^ Data suggest that the institutional safety climate element consistently scores the lowest compared with other elements, with no significant differences between high- and low-income countries.^7 8^

Within the broader safety and quality literature, a safety culture has been described as the way patient safety is thought about and implemented within an organisation and the structures and processes in place to support this.^9^,^11^ It has also been highlighted as an influencer of care processes and outcomes.^11^ In the context of hand hygiene, the institutional safety climate refers to the organisational environment and perceptions of patient safety issues in the healthcare setting where hand hygiene improvement is considered as a high priority.^12 13^ To date, a limited number of studies have demonstrated the inter-relationship between the safety culture, IPC processes (including hand hygiene) and improved patient outcomes.^14 15^ Improving the organisational safety climate has been associated with enhanced hand hygiene compliance and a reduction in healthcare-associated infection (HAI),^16^,^20^ including central line-associated bloodstream infections.^21 22^

The paucity of studies on the connection between the safety climate/culture change and hand hygiene presents a challenge, particularly given the lack of a uniform definition of what this entails across various healthcare settings. The aim of this study was to reach an international consensus among stakeholders from diverse backgrounds, including clinicians, policy-makers, researchers and funders, on a research agenda highlighting essential priorities for enhancing an institutional safety climate within the framework of a multimodal hand hygiene improvement strategy.

## Methods

A multimethod, multiphase approach was adopted to establish expert-derived research agenda priorities related to the role of institutional safety climate in hand hygiene improvement. The Delphi consensus methodology, as described by Jones and Hunter,^23^ was employed and comprised five stages as described below: (1) literature review and expert identification; (2) expert panel meeting; (3) round 1 survey; (4) round 2 survey and (5) final review. The study protocol adhered to recommendations for the Conducting and REporting of DElphi Studies.^24^ While the conventional Delphi technique includes open-ended questions in the initial questionnaire and continues with as many rounds as necessary to attain consensus, we adopted a modified Delphi study, in line with other studies, featuring two alterations: (1) conducting a literature review and evidence mapping to develop an initial list of research priorities which were embedded in an electronic survey for the Delphi process and (2) establishing a predetermined number of survey rounds (two).^25^,^31^

The ‘institutional safety climate’ terminology used in this study refers to the environment and perceptions of patient safety issues at the healthcare setting and facility, in which hand hygiene improvement is considered a high priority.^12 13^

### Stage 1: supporting literature review and expert identification

To support this project, a literature review structured in two parts was conducted, to understand the evidence base, consider evidence gaps and develop research statements relevant to the connection between hand hygiene and institutional safety climate within healthcare settings. The initial analysis focused on ‘Hand hygiene: a Handbook for Medical Professionals’,^12^ which builds on the WHO guidelines on hand hygiene in healthcare.^13^ Subsequently, a literature review modelled on scoping review methods^32^ was conducted using the following keywords in combinations with the Boolean terms AND and OR: safety (AND) (culture OR climate)) (AND) hand hygiene (OR) handwashing (OR) hand sanitization (OR) hand rub (OR) hand clean (OR) hand decontamination (OR) hand gel. Searches were performed in Medline, Embase, Global Health, Web of Science and Cochrane Collaboration databases from inception until January 2022, resulting in 46 articles being included in the review. The findings from this review laid the groundwork for developing a list of research priorities which were then reviewed and discussed by the Safety Climate Working Group of the WHO Technical Advisory Group (TAG) on hand hygiene in healthcare and IPC research.^33^

By employing a non-probability purposive sampling technique to ensure that participants met the inclusion criteria, individuals were recruited from all six WHO regions (Africa, the Americas, the Eastern Mediterranean, Europe, Southeast Asia and the Western Pacific), as well as across all World Bank economic income levels.^34^ We used three strategies to identify and recruit participants. First, within the TAG, all Safety Climate Working Group members were invited to participate in the Delphi study. Second, we identified individuals with expertise and established reputations in the field of hand hygiene research and institutional safety climate and culture change through literature searches and via networks of WHO stakeholders. Finally, we reached out to WHO regional offices to identify potential participants from regions where the required participation had not yet been met. Research team members did not participate in the Delphi surveys.

### Stage 2: expert panel meeting

The list of research priorities on institutional safety climate in the context of a multimodal hand hygiene improvement strategy comprised 36 statements categorised into 5 thematic areas: (1) safety climate/culture change; (2) personal accountability for hand hygiene; (3) leadership; (4) patient participation and empowerment and (5) religion and traditions.^33^ The list was sent to participants, who were then invited to attend a meeting of TAG members, and an expert panel to review and provide feedback on the research priorities or suggest additional priorities. The Working Group chair and facilitator led the discussion by revising priorities, rephrasing, removing duplicates and merging similar topics. Feedback from this process further consolidated research priorities which were then used for this study’s stage 3 Delphi round 1 survey.

### Stage 3: Delphi survey round 1

The revised research priorities were embedded in the electronic survey of the Delphi process. The survey and platform were piloted to assess the content and flow, clarity of instructions and ease of use. This survey was administered online via Welphi Manager software.^35^ In total, the survey comprised 36 statements, to which experts were requested to indicate their level of agreement, using a 5-point Likert scale ranging from 1 (‘totally disagree’) to 5 (‘totally agree’), with an additional option of ‘unable to rate’. They were instructed to evaluate the significance of each research priority by considering the dimensions of impact/significance, cost-effectiveness and feasibility of the research.^33^ The primary outcome was to attain consensus on the research priorities to be included in the final hand hygiene research agenda list. The survey was emailed to 57 experts, who were instructed to score each research priority according to their assessment of the need for further evidence in the field. Experts were invited to provide free-text comments for each priority, including suggestions for the revision of the proposed priorities and proposals for additional items for consideration.

### Stage 4: Delphi survey round 2

Participants who completed round 1 were invited to participate in round 2. Results of round 1 were analysed and shared with participants, including a visual representation of the distribution of scores from all participants, together with the participant’s own score, using the same Likert scoring scale as described in round 1. The identities of the individuals were kept anonymous. Participants were asked to consider the responses of other respondents and review their scores, either confirming or modifying them. Research priorities that had not reached consensus in the initial round were presented for re-evaluation and scoring. The Delphi surveys (rounds 1 and 2) were completed between 1 March 2022 and 26 April 2022. Each round was open for approximately 10 days, and the participants received two reminder emails to complete the survey.

### Stage 5: final review

A final consultation meeting of the WHO TAG on Hand Hygiene in Healthcare experts and the WHO Secretariat resulted in the review and consolidation of research priorities into a comprehensive research agenda.

### Data analysis

Descriptive statistics were used to describe the participants’ demographic characteristics and their responses to each statement in both rounds. Results were presented in a Microsoft Excel (Microsoft, Redmond, USA) spreadsheet format for each Delphi survey. Consensus was defined as >70% of the participants agreeing/strongly agreeing with a statement in each round. ‘Disagreement’ was defined as 35% or more of the responses falling within both extreme ranges on the Likert scale. This level of agreement has been considered appropriate in previous Delphi studies.^36^,^46^

All ‘unable to rate’ responses were excluded from the group response to ensure that the reported percentage agreement or disagreement for each statement represented the consensus among only those who knew the answer.^33^ The mean, SD and proportion rating were calculated for each item outcome. Analyses were conducted using IBM SPSS V.25.

## Results

Of the 57 experts invited to participate in the Delphi study, 50 completed round 1 (88% response rate) and 48 of 50 completed round 2 (96% response rate). There were 12% non-responders, and a dropout rate of 4%. Table 1 provides an overview of the respondents’ demographic characteristics. There was a higher representation of participants from high-income (51%) vs upper-middle-, lower-middle- and low-income countries (19%, 16% and 9%, respectively) (table 1). Participants came from a wide range of disciplines, including IPC (72%), infectious diseases (46%), microbiology (28%), patient safety and quality (49%) and research and academia (46%); 40% had over 15 years of experience in their respective fields. Round 1 was completed between 8 and 14 April 2022 and round 2 took place between 16 and 26 April 2022.

**Table 1:** Characteristics of the expert panel in the Delphi survey round 1

	Round 1 N=57 (%)
WHO region	
Africa	7 (12.3)
Americas	12 (21.1)
Eastern Mediterranean	5 (8.8)
Europe	11 (19.3)
South-East Asia	12 (21.1)
Western Pacific	7 (12.3)
Unknown	3 (5.3)
World Bank income level	
High	29 (50.9)
Upper-middle	11 (19.3)
Lower-middle	9 (15.8)
Low	5 (8.8)
Unknown	3 (5.3)
Area of expertise	
Infection prevention and control	41 (72)
Hand hygiene	35 (61.4)
Infectious disease	26 (45.6)
Microbiology	16 (28.1)
Patient safety quality	28 (49.1)
Academic research	26 (45.6)
Guideline development	23 (40.4)
Water, sanitation and hygiene	6 (10.5)
Other	9 (15.8)
Years of experience in the current expertise	
5–10	9 (15.8)
10–15	7 (12.3)
>15	23 (40.4)
Unknown	18 (316)

Table 2 shows the Delphi research statements, categorised into five thematic areas. A total of 36 research priority statements were initially identified by the Safety Climate Working Group of the WHO TAG on Hand Hygiene in Healthcare and the WHO Secretariat, and discussed in an expert panel meeting before launching the Delphi survey. Following discussion, some statements were rephrased to improve clarity. The number of statements in which consensus was achieved improved from rounds 1 to 2 (online supplemental table 1). In round 1, consensus was achieved for 75% (n=27) of the 36 research statements. Table 3 presents the statements that did not achieve consensus in round 1 and were included in round 2 and statements that finally did not reach consensus. The nine statements that did not achieve consensus were related to the following domains: safety climate/culture change (one item), personal accountability for hand hygiene (two items), patient participation and empowerment (three items) and religion and traditions (three items) (table 3; online supplemental table 2).

**Table 2:** Results of Delphi survey round 1

	Agree (%)	Neutral (%)	Disagree (%)	Unable to rate (%)	Mean±SD
Safety climate/culture change					
Influence of different cadres of the health workforce on the institutional safety climate	86	10	4	0	1.72±0.8
Perspectives of different cadres of health workers towards an institutional safety climate	84	14	2	0	1.86±0.72
The relationship between a healthcare facility’s safety and quality climate/culture and the culture related to hand hygiene (and IPC)	90	6	2	2	1.51±0.7
The influence of a healthcare facility’s safety and quality climate/culture on hand hygiene practices during outbreaks/emergencies/pandemics	82	14	4	0	1.76±0.84
Role of media (mainstream and social media) in shaping/influencing an institutional safety climate and hand hygiene improvement	68	24	8	0	2.14±0.96
The role of hand hygiene campaigns (including promotional messages and campaign communications, reminders in the workplace) in shaping/influencing a sustained institutional safety climate	86	8	6	0	1.74±0.91
Personal accountability for hand hygiene					
Best methods for measuring personal accountability (health workers are accountable for their hand hygiene behaviour)	84	10	6	0	1.72±0.94
Relationship between the training of individual health workers and personal accountability for hand hygiene improvement	72	16	10	2	1.96±1.05
Influence of an enabling environment (built environment, materials and equipment for hand hygiene) on personal accountability for hand hygiene	86	12	0	2	1.59±0.7
Relationship between different methods for the monitoring and feedback of hand hygiene performance and personal accountability for hand hygiene	84	12	2	2	1.57±0.78
Impact of different types of appraisal/reward systems/incentives (including financial) on personal accountability for hand hygiene	80	12	6	2	1.8±0.88
Relationship between individual health worker perceptions of hand hygiene and personal accountability	72	22	6	0	2.06±0.86
Influence of hand hygiene champions/role models (people providing the example/advocating for the causes of patient safety and hand hygiene standards) on personal accountability for hand hygiene	74	18	6	2	1.92±0.97
Factors that influence the development (eg, training, mentoring, attitudes, beliefs, values) of an effective hand hygiene champion	66	26	6	2	2.0±0.95
Relationship between a leadership approach that demonstrably values hand hygiene (eg, allocates resources, plans, evaluates, contextualises, refreshes strategies for hand hygiene improvement) and the personal accountability of individual health workers	88	8	2	2	1.59±0.73
Influence of different institutional social networks on personal accountability for hand hygiene	60	34	6	0	2.24±0.93
Leadership					
Effectiveness of a leadership approach that demonstrably values hand hygiene through a multimodal improvement strategy to improve the overall institutional safety climate	82	12	4	2	1.65±0.85
Most effective governance structures for shaping/influencing an institutional safety climate that supports hand hygiene	86	10	0	4	1.58±0.67
Barriers and drivers at the leadership/management and individual level to institutionalise hand hygiene as a priority	86	10	4	0	1.6±0.82
Influence of IPC/hand hygiene training targeted at hospital leadership on an institutional safety climate	82	12	6	0	1.76±0.88
Direct relationship between leadership support for hand hygiene and hand hygiene improvement/performance	82	14	2	2	1.69±0.79
Leadership factors influencing an institution’s commitment to hand hygiene improvement	82	14	4	0	1.74±0.84
Relationship between a national IPC programme (according to the requirements laid out in the WHO national-level core components) and its relevance and influence on institutional safety climate	76	20	4	0	1.88±0.93
Patient participation and empowerment					
Relationship between patient participation/empowerment strategies and the establishment of an institutional safety climate that values hand hygiene	86	8	6	0	1.72±0.92
Factors that motivate decision-makers/senior managers to involve patients within institutional strategies for improving hand hygiene	70	24	6	0	1.98±0.93
Impact of patient participation/empowerment on hand hygiene improvement including the influence of patient participation on hand hygiene improvement at different implementation stages (eg, programme design and point of care)	80	18	2	0	1.84±0.86
Role and impact of visitors and informal caregivers in hand hygiene improvement	65	29	6	0	2.19±0.91
Perceptions of service users and patients towards an institutional safety climate and its impact on hand hygiene standards	68	20	10	2	2.15±0.98
Relationship between national culture characteristics (especially dimensions of power distance and uncertainty avoidance) and patient participation/empowerment in hand hygiene initiatives	70	26	2	2	2.02±0.8
Most effective methods of patient participation/empowerment to improve institutional hand hygiene practices	84	12	4	0	1.62±0.91
Barriers and facilitators of patient participation/empowerment in hand hygiene interventions	80	14	6	0	1.82±0.95
Underlying ethical considerations in the development of a patient participation/empowerment strategy as part of an overall approach to hand hygiene improvement	58	24	12	6	2.23±1.17
Religion and traditions					
Influence of different religions and traditions on hand hygiene improvement strategies, including as barriers and facilitators	68	22	10	0	2.26±0.91
Relationship between religious beliefs, practices and traditions and institutional safety climate that influences hand hygiene	60	28	10	2	2.29±0.9
Influence and impact of wider societal norms (including national and organisational culture, religion and traditions) on the institutional safety climate that influences hand hygiene	78	18	0	4	1.83±0.72
Influence of religion and traditions on acceptance and use of alcohol-based hand hygiene products	52	24	22	2	2.55±1.21

IPC, infection prevention and control.

**Table 3:** Results of Delphi survey round 2, including statements not reaching consensus

	Agree (%)	Neutral (%)	Disagree (%)	Mean±SD
Safety climate/culture change				
Role of media (mainstream and social media) in shaping/influencing an institutional safety climate and hand hygiene improvement	75	19	6	2.12±0.78
Personal accountability for hand hygiene				
Factors that influence the development (eg, training, mentoring, attitudes, beliefs, values) of an effective hand hygiene champion	79	15	6	1.75±0.98
*Influence of different institutional social networks on personal accountability for hand hygiene*	*67*	*31*	*2*	*2.06±0.82*
Patient participation and empowerment				
Role and impact of visitors and informal caregivers in hand hygiene improvement	71	21	8	2.08±0.9
Perceptions of service users and patients towards an institutional safety climate and its impact on hand hygiene standards	81	10	6	1.99±0.84
*Underlying ethical considerations in the development of a patient participation/empowerment strategy as part of an overall approach to hand hygiene improvement*	*61*	*23*	*12*	*2.11±1.16*
Religion and traditions				
*Influence of different religions and traditions on hand hygiene improvement strategies, including as barriers and facilitators*	*69*	*21*	*10*	*2.3±0.87*
*Relationship between religious beliefs, practices and traditions and institutional safety climate that influences hand hygiene*	*62*	*21*	*15*	*2.42±0.87*
*Influence of religion and traditions on acceptance and use of alcohol-based hand hygiene products*	*52*	*21*	*25*	*2.57±1.2*

Italicised statements indicate areas not reaching consensus.

Of these, four achieved consensus, while five statements did not reach consensus in round 2 (one each in the domains of personal accountability and patient participation and empowerment, and three in the religion and traditions domain) (table 3). Overall, in round 2 consensus was achieved for 86% (n=31) of the 36 items. By round 2, 100% consensus was achieved for the domains of safety climate/culture (n=6) and leadership (n=7); the lowest level of consensus was 25% for religion and traditions (n=4).

For the ‘safety climate/culture’ domain, consensus achieved highlighted the need to research the ‘safety climate during outbreaks/emergencies’ (82%, mean±SD: 1.76±0.84); ‘an institution’s safety climate and hand hygiene improvement’ (90%, mean±SD: 1.51±0.7) and ‘the role of hand hygiene campaigns’ (86%, mean±SD: 1.74±0.91) and ‘media/social media’ (75%, mean±SD: 2.12±0.78) in influencing and sustaining an institutional safety climate. For the ‘leadership’ domain, further research should endeavour to evaluate effective leadership (82%, mean±SD: 1.65±0.85) and governance structures (86%, mean±SD: 1.58±0.67) in shaping and reinforcing a culture that supports hand hygiene improvement; leadership drivers influencing an institution’s commitment to prioritise hand hygiene improvement (86%, mean±SD: 1.58±0.67) and the impact of IPC training for hospital leadership (82%, mean±SD: 1.76±0.88). For the ‘patient participation and empowerment’ domain, consensus was attained for eight (89%) of the nine statements, highlighting effective methods for patient empowerment (84%, mean±SD: 1.62±0.91) and its impact to enhance institutional hand hygiene (80%, mean±SD: 1.84±0.86) and drivers of patient participation in hand hygiene interventions (80%, mean±SD: 1.82±0.95). The remaining statement describing the need for ethical considerations in a patient participation/empowerment strategy could not be agreed on by the expert panel. Results for the ‘personal accountability for hand hygiene’ domain emphasise the need to evaluate the role of an ‘enabling environment and effective monitoring and feedback mechanism in enhancing personal accountability for hand hygiene’ (86%, mean±SD: 1.59±0.7) and ‘leadership approaches in influencing personal accountability of health workers’ (88%, mean±SD: 1.59±0.73). For the ‘religion and traditions’ domain, the only statement attaining consensus was the influence and impact of wider societal factors in shaping institutional practices, including national and organisational culture, religion and traditions (78%, mean±SD: 1.83±0.72).

### Participant comments and final review

**Figure 1:**
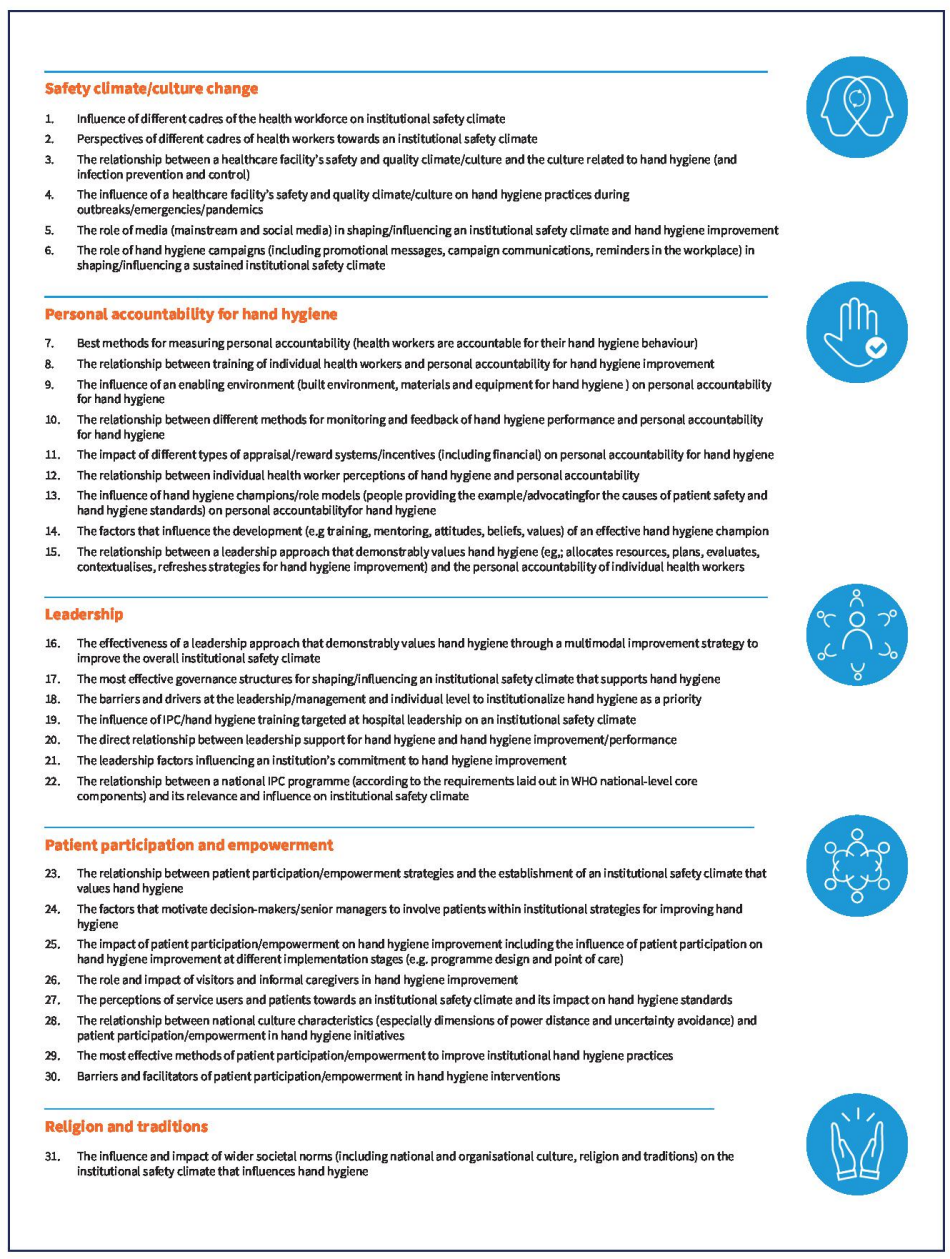
Final research priorities achieved for enhancing an institutional safety climate within the framework of a multimodal hand hygiene improvement strategy categorised into five thematic areas following consensus with experts. IPC, infection prevention and control.

Participants provided free-text comments, with 120 comments across both rounds (round 1, n=87; round 2, n=33). These comments added a qualitative dimension to the results and were carefully reviewed by the research team. The comments in round 1 resulted in direct additions, subtractions or rephrasing of priority statements that progressed to round 2. The comments fell into a number of categories, including opinion-based, general reflections, those suggesting clarifications and those provided to justify scoring. Examples are summarised in online supplemental table 3A,B. The comments included are those that most pertained to the overall aim of the two rounds of prioritising the actual statements. Following a final review and consultation meeting, expert members of the WHO TAG on Hand Hygiene in Healthcare and the WHO Secretariat agreed to a refined set of 31 research priorities listed according to the five pre-established overarching thematic categories (figure 1).

## Discussion

This Delphi study identified 31 research priorities, focusing specifically on the institutional safety climate for hand hygiene improvement. Consensus was achieved after two rounds of review by a panel of 57 international experts specialising in IPC. The survey reached consensus on 86% of the proposed research statements across the five overarching domains. To the best of our knowledge, this is the first comprehensive effort to date to identify a cohesive set of research priorities that aims to address HAI and antimicrobial resistance as an institutional patient safety problem or improvement of hand hygiene in healthcare facilities.

The following priorities and cross-cutting themes emerged from the five thematic categories, highlighting key areas for research: effective leadership and governance structures for shaping an institutional safety climate that supports hand hygiene initiatives; the influence of wider societal norms such as national, organisational culture, religion and traditions on the institutional safety climate; the impact of personal accountability and the built environment on the behaviours of healthcare professionals towards hand hygiene; understanding how media and social media can be leveraged to stimulate behaviour change; role of a healthcare facility’s safety and quality climate on hand hygiene during outbreaks, emergencies and pandemics and the contribution of patients and civil society to hand hygiene improvement.

Experts agreed on the importance of further exploring the relationship between leadership support, governance structures and hand hygiene improvement to enhance the institutional safety climate. Furthermore, the role that societal norms (including national, organisational culture, religion and traditions) play in shaping institutional safety climate underscores the need for a deeper understanding of the frameworks that effectively promote a culture of safety and quality in which hand hygiene improvement is considered a priority.^20^ Variability in HAI rates and hand hygiene compliance has highlighted the potential impact of organisational culture, governance structure and leadership engagement on IPC efforts.^2047^,^50^ Wider societal norms have been linked to variations in healthcare outcomes, such as the rates of methicillin-resistant *Staphylococcus aureus* infections and unjustified antibiotic use in European countries.^25 49^

The literature on quality management consistently points to leadership and a supportive culture as fundamental for the successful implementation and sustainability of effective interventions.^51^ IPC literature also echoes the importance of leaders in establishing a favourable organisational culture that supports IPC practices, crucial for overall care quality.^52 53^ Leadership is central to effective hand hygiene improvement strategies as part of the WHO MMIS, where leadership engagement has been linked to sustained improvements in compliance.^54^ However, there remains a gap in identifying clear models for leadership and governance for patient safety that set standards and behaviours for leaders where hand hygiene improvement is considered a high priority at an organisational level.^55^ Huis *et al*^56^ in a cluster-randomised controlled trial compared a social cognitive theory-based team and leader-directed strategy against a conventional approach involving system change, education, reminders and feedback to improve hand hygiene compliance. Hand hygiene compliance in the experimental group receiving additional leadership and social influence interventions significantly improved, from 20% to 53% and was sustained over time. The authors underscored the role of leadership, social influence and team dynamics in effective behavioural change.

The expert panel also agreed on the importance of identifying the most effective methods for measuring personal accountability, the impact of appraisal/reward systems and the influence of champions/role models on personal accountability. For example, there is considerable scope for enhancing health workers’ accountability for their performance standards. In an interrupted time series analysis, Talbot *et al*^27^ reported a sustained and high level of hand hygiene compliance following the introduction of a direct observation programme combined with goals and incentives and by promoting individual and group accountability. By engaging leadership through a formal accountability structure and offering institutional financial incentives, nursing and physician leadership were motivated to create a culture of sustained hand hygiene compliance (75% at the intervention phase compared with 52% at baseline and 89% during the ‘active accountability’ phase). When considering personal accountability for hand hygiene, experts agreed that there was a need for organisations to evaluate ‘the influence of an enabling environment (built environment, materials and equipment for hand hygiene) on personal accountability for hand hygiene’.

Consensus was reached on the research priority ‘role of media and social media in influencing an institutional safety climate and hand hygiene improvement’. Of note, as the 2009 WHO guidelines^13^ on hand hygiene preceded the social media age, the use of mass media was only evoked in the context of campaigns rather than as an adjunct to efforts to improve the safety climate. Thus, the current priority centred on the role of social media in shaping or influencing the safety climate and hand hygiene improvement could lead to novel research involving entities outside the healthcare arena (online supplemental table 4). Social media has been reported to play a valuable role in safeguarding populations against harm caused by HAIs and in disseminating evolving information during the COVID-19 pandemic. As such, it is deemed worthy to further explore this role.^57 58^

The panel recommended that it is essential to conduct research on the influence of a healthcare facility’s safety and quality climate on hand hygiene during outbreaks, emergencies and pandemics, given the recent global health crisis. The COVID-19 pandemic has highlighted significant gaps in our understanding of the determinants of consistent hand hygiene practices during critical periods. While some studies^59^ reported unchanged or even reduced compliance rates during the pandemic, others noted an initial improvement at the beginning of the pandemic, followed by a decline.^60 61^ Addressing these gaps requires a focused evaluation of the behavioural determinants of hand hygiene in practice. By understanding these determinants, targeted interventions can be developed to improve hand hygiene compliance and to strengthen the institutional safety and quality culture and preparedness for future global health challenges.

Consensus was reached on research priorities centred on patient participation, particularly to explore how it influences the institutional safety climate and the interaction between national cultural characteristics and patient/civil society participation in hand hygiene initiatives.^1547^,^54 56 62^ Despite the recognised value of patient involvement in shaping healthcare systems through consultation and feedback, implementation remains scarce.^65^ While patient engagement is crucial for fostering a culture of safety in healthcare, the most effective methods for optimising such engagement to improve hand hygiene practices are not well-defined.^66^,^69^ Although patients may have a potentially important role in reminding health workers to perform hand hygiene, they may view patient participation as unrealistic.^66 70 71^ In a cluster, randomised controlled trial on the effect of two additional interventions (enhanced performance feedback or enhanced performance feedback plus patient participation), Stewardson *et al*^68^ observed an institution-wide improvement with hand hygiene compliance of health workers (65%–74%) between the baseline and intervention periods. However, patient involvement is often considered impractical, and hand hygiene practices continue to face implementation challenges.

### Research agenda: how can this be implemented?

The utility and relevance of our findings can be considered within the current global, national, subnational and health facility-level context for IPC implementation. This research agenda concurs with WHO’s global strategy on IPC^72^ and associated global action plan and monitoring framework^73^ requesting all countries to develop a country-specific national research agenda and priorities for IPC, including a multisectoral and multidisciplinary approach. Indeed, there is a global target that over 80% of countries have a national IPC research agenda by 2030. To meet this, WHO advocates the use of a five-step cycle of implementation based on implementation and quality improvement science.^74^ These 31 research priorities for hand hygiene will go some way to support countries in prioritising, funding, and implementing research projects on IPC in selected facilities, according to local priorities. Qualitative studies are required to understand the factors affecting IPC interventions’ success or failure, particularly the intangible components that shape the attitudes, beliefs and values of clinicians, so that they consistently perform tasks the way they know they should. The ‘safety culture’, including improved communication and teamwork, is part of these elements and requires further investigations to be better understood. Online supplemental table 4 provides an initial list of possible research questions according to each priority, and outlines potential stakeholders and funding sources, including indicative examples of possible research designs.

### Strengths and limitations

This study is the first to achieve a consensus on research priorities related to the institutional safety climate in hand hygiene improvement within healthcare settings. A key strength lies in the global engagement of internationally renowned experts from diverse backgrounds in dialogue to identify how to address the key knowledge gap in the institutional safety climate for hand hygiene improvement.

Additionally, it was informed by an extensive literature search and achieved a high response rate (88%, round 1 and 96%, round 2) and a high retention rate between both survey rounds, ensuring that the final consensus on the future research agenda for hand hygiene reached by the experts was widely accepted. The current literature provides varying thresholds for acceptable agreement among participants in Delphi studies. We presented a threshold of 70% in each round, which was within the range of agreement rate values suggested in the literature (51%–80%). Our Delphi survey protocol included only two rounds of consensus-building, which might be perceived as a limitation, although this modified approach is supported by evidence. Criticisms of the Delphi method include the perception that it forces consensus and that it is weakened by not allowing participants to discuss the issues raised. We attempted to address this through a participatory and iterative process in parallel with the actual Delphi exercise through multiple meetings of the TAG and the Safety Climate Working Group. While our sample achieved a reasonable spread of countries internationally, some regions and low-income countries were less represented by the expert panel. Finally, the views of more diverse stakeholder groups, such as healthcare facility leadership, health workers and patients, should be sought on this topic.

## Conclusion

This study has produced an internationally endorsed, expert-led research agenda focused on the role of the institutional safety climate in enhancing hand hygiene. Progress in this field will depend on global collaborative efforts by healthcare and academic organisations to guide future research. This agenda serves as a valuable tool for research networks, clinicians, policy-makers, quality improvers and national and international funding bodies. Addressing these priorities will be essential in advancing the global agenda for hand hygiene improvement and IPC, ultimately ensuring safe and high-quality healthcare for all, free from avoidable infections.

## Supplementary material

10.1136/bmjqs-2024-017162 online supplemental file 1

## Data Availability

Data are available on reasonable request.
